# Social isolation in older adults with type 2 diabetes mellitus—a path analysis

**DOI:** 10.3389/fpubh.2025.1562186

**Published:** 2025-07-25

**Authors:** Jing Wang, Xiaoyan Bai, Keke Lin, Chao Sun, Quanying Wu, Ruiting Zhang, Yu Liu

**Affiliations:** ^1^School of Nursing, Beijing University of Chinese Medicine, Beijing, China; ^2^Nursing Department, Beijing Hospital, National Center of Gerontology, Institute of Geriatric Medicine, Chinese Academy of Medical Sciences, Beijing, China; ^3^School of Traditional Chinese Medicine, Beijing University of Chinese Medicine, Beijing, China

**Keywords:** type 2 diabetes, aged, social isolation, influencing factors, path analysis

## Abstract

**Background:**

Due to aging, the use of antidiabetic drugs, and dietary restrictions following a diagnosis of type 2 diabetes mellitus (T2DM), the social interactions of older adults with T2DM are often limited. As a result, this population experiences a higher incidence of social isolation than the general older adult population. This study aims to analyze the prevalence and influencing factors of social isolation among older adults with T2DM using a structural equation model.

**Patients and methods:**

A cross-sectional study was conducted between January and November 2023. A total of 496 older adults with T2DM were recruited from hospitals or community health service centers in Beijing to investigate their social isolation status and related factors. The Lubben Social Network Scale-6, along with related scales, was used for data collection. The effects of different factors on social isolation were determined using a path analysis.

**Results:**

Among 496 older adults with T2DM, 227 reported social isolation, resulting in a prevalence rate of 45.77%. Activity of daily living, cognitive function, loneliness, exercise management, smoking, social support, and social participation are all directly related to social isolation. Additionally, six factors—activity of daily living, loneliness, depression, diet management, blood glucose monitoring, and social support—were related to social isolation through social participation.

**Conclusion:**

The incidence of social isolation among older adults with T2DM is high. For them, activities of daily living, loneliness, and social support are significant factors in their social isolation since they are directly or indirectly related to social isolation. Meanwhile, diabetes self-management, such as diet management, exercise management, blood glucose management, and smoking, is directly or indirectly related to social isolation. For older adults with T2DM, the important intermediary role of social participation between the factors and social isolation should be given due attention.

## 1 Introduction

With aging, the proportion of the older adult population with diabetes in China has gradually increased. Approximately 30% of Chinese older adults have diabetes, and 95% of them suffer from type 2 diabetes mellitus (T2DM) (1). Social isolation is a well-established risk factor for adverse health and psychological outcomes. A recent meta-analysis revealed that socially isolated individuals have a 1.88-fold higher risk of developing T2DM compared to non-isolated individuals (2). After being diagnosed with diabetes, some patients avoid communicating with others because they are worried that their diet is challenging to control, or it is inconvenient to use hypoglycemic drugs when going out. Moreover, those with diabetes complications, such as diabetic foot, are forced to reduce social activities due to physical restrictions (3). These situations may increase the difficulty of maintaining the social network of older adults with T2DM. Therefore, those with diabetes are at high risk of social isolation. Moreover, aging increases the risk of social isolation. In China, people aged 60 or above are classified as older adults (4). Due to retirement or changes in family structure (i.e., the passing away of a spouse or children away from home), older adults’ social network tends to shrink, and the risk of social isolation increases accordingly. Compared with older adults in Western society, the social network size of older adults in China is smaller, with a higher proportion of family members, and their social network is mainly based on kinship (5). However, since the 1970s, the continuously declining birth rate and intensified population mobility have led to a trend toward smaller households in China, which may increase the risk of social isolation among older adults in China (5). In 2019, a survey in Beijing, China, showed that the incidence of social isolation among older adults with T2DM was 22.7% (6).

The World Health Organization (WHO) defines health not merely as the absence of disease or infirmity, but as a state of complete physical, mental, and social wellbeing (7). Social isolation adversely affects both physical and mental health, contributing to suboptimal health status, increased incidence of physical and mental disorders, and ultimately higher mortality rates. At the societal level, it can drive detrimental shifts in social structure and impede socioeconomic and cultural development. Consequently, social isolation is widely recognized as a critical global public health challenge (8).

Social isolation is a state of disconnection from meaningful connections with others or society, which can manifest as a lack of marital status (unmarried, widowed or divorced), failure to participate in any social groups, community organizations, or interest-based activities, a lack of close ties with relatives and friends, or an emotional support network (9). This condition has significant implications for health and wellbeing. It is associated with negative emotional states (e.g., anxiety and depression), cognitive decline (10, 11), and unhealthy behaviors such as reduced physical activity and poor treatment adherence—factors that undermine effective diabetes self-management (12, 13). Moreover, poor self-management in diabetic patients leads to significant blood glucose fluctuations, which may further impair social interactions, reduce social network size, and exacerbate isolation (13). This cyclical relationship creates a self-perpetuating vicious cycle, worsening both health outcomes and social connectivity.

Social isolation in older adults with diabetes may be associated with self-management, physical activity, blood glucose fluctuation, and physical function (14), but few studies have reported the factors affecting social isolation in older adults with diabetes in China. In addition, existing studies focus on single or scattered factor categories related to social isolation, without exploring the relationships between these factors. This makes it difficult to provide a strong reference for intervention in social isolation for older adults with diabetes. Therefore, it is of great significance to explore the factors associated with social isolation and their interplay in older adults with diabetes.

Wenger’s conceptual framework of social networks for older adults is mostly used for studying social networks (15). Social network refers to the mutual relationship between individuals and their family members, relatives, friends, and other interactive members. Social isolation is a special state of social network characterized by a low level of social interaction. In the framework, the structural characteristics of social network (i.e., frequency of contact, closeness, and distance) in older adults are impacted by various factors such as personal characteristics, external environment characteristics, and social support. Social support refers to the degree of spiritual and material connection between individuals and various aspects of society, including relatives, friends, colleagues, partners, and social organizations such as family units (16). Under the guidance of Wenger’s conceptual framework and combined with the related factors of social isolation of patients with diabetes shown in literature (6), we speculate that the social isolation of the older adults with T2DM are related to individual characteristics (visual impairment, hearing impairment, activity of daily living, cognitive function, self-management, depression, loneliness, etc.), external environment characteristics (family function and social participation, etc.), social support, and the use of formal services (community health service utilization).

Social participation refers to the behavior and process of an individual actively interacting with society and integrating into the family and social environment (17). Appropriate social participation increases the contact between individuals and others, expands their social network size, and helps individuals to maintain good social relationships. Conversely, impaired social participation ability reduces social participation and aggravates the degree of social isolation of the older patients (17). For older adults with T2DM, it was reported that social participation was associated with self-management, social support, loneliness, and the ability to perform daily living activities (18). From this inference, social participation may be a mediating variable in the model of social isolation-related factors; that is, other factors may indirectly affect the occurrence of social isolation through social participation.

This study used questionnaires to investigate the factors related to social isolation in 496 older adults with T2DM and conducted path analysis using a structural model to elucidate the mechanism of social isolation in these older adults and provide a basis for developing targeted interventions. We hypothesized that, after controlling for general demographic factors, 16 factors such as visual impairment, hearing impairment, self-management, diabetes self-management activities (such as diet, medication, exercise, blood glucose monitoring foot care, and smoking), social support, social participation may directly affect the occurrence of social isolation in the older adults with T2DM; meanwhile, some factors may affect the social isolation indirectly through social participation.

## 2 Materials and methods

### 2.1 Study design and sample

From January to November 2023, a cross-sectional study was carried out with a convenience sample of older adults with T2DM at the China–Japan Friendship Hospital, the Third Affiliated Hospital of Beijing University of Chinese Medicine, the Beijing Hospital, and the Fangzhuang Community Health Service Center.

The inclusion criteria for this study are as follows: ① Being ≥60 years old, ② having a diagnosis of T2DM (according to the diagnostic criteria of diabetes issued by the Chinese Medical Association (19)) for more than 1 year, ③ Chinese nationality, ④ score on the Mini Mental State Examination (MMSE) > 20 points. The exclusion criteria for this study are as follows: ① Having acute complications of diabetes, ② Having serious diseases caused by non-diabetes (such as a tumor), ③ Having a severe mental disorder or language communication barriers.

According to the sample size calculation method of the structural equation model (20), the sample size needs to be at least 10 times the number of observed variables. In this study, there were 31 observed variables in the study, and considering the possible invalid questionnaire, the sample size was increased by 10%. Therefore, the theoretical sample size was at least 341 cases. This study included a total of 496 samples.

### 2.2 Measurement

#### 2.2.1 Lubben social network scale-6 (LSNS-6)

LSNS-6 is a brief version of the social network scale compiled by Lubben (21), which identifies an individual’s social isolation based on frequency and intimacy with their friends and family. It includes family and friend dimensions, each with six questions. The score for each question is 0–5. A total score lower than 12 indicates social isolation, and a score of less than 6 for each dimension indicates isolation in this dimension. Lower scores indicate a higher level of social isolation. The Chinese version of LSNS-6 and its subscales have good reliability and validity (22). Cronbach’s *α* coefficients for the total scale, and family and friend subscales, in this study were 0.856, 0.917, and 0.915, respectively.

#### 2.2.2 Scale sets on factors related to social isolation

1. General Information Sheet: This is a self-designed sheet used to investigate part of the influencing factors of social isolation. The sheet includes demographic data (gender, age, education level, marital status, etc.), disease-related data (number of chronic diseases, fasting blood glucose value, diabetic complications, visual function, hearing function, etc.), and social data (living status, community transportation conditions, and health service utilization). Patients with poor vision or blindness due to cataract, glaucoma, and diabetic retinopathy were regarded as having visual impairment. Those with tinnitus, unclear hearing, and the use of hearing aids were regarded as having hearing impairment. The assessment of health service utilization refers to the assessment of patients’ attitudes and use of healthcare services, that is, the types of care services that older adults were aware of and/or received and were willing to accept.
2. Activity of Daily Living Scale (ADLs): ADLs is composed of the physical self-maintenance scale and instrumental activities of daily living scale to assess an individual’s physical self-care ability (such as walking and eating independently) and instrumental daily living ability (such as shopping, cooking, and transportation). It has 14 items, which are measured on a 4-point Likert scale (ranging from “fully capable” ~ to “completely impossible”). The higher the total score, the poorer the living ability (23). ADLs demonstrated good applicability in the Chinese older adult population, with Cronbach’s *α* of 0.894 (24).
3. Mini-Mental State Examination (MMSE): The MMSE was used to evaluate an individual’s cognitive function in five aspects: orientation (time and place), immediate memory, attention and computing power, delayed memory, and language, totaling 30 points. A total score of no less than 27 points indicates normal cognition, while 21–26 points indicates mild cognitive impairment, 10–20 indicates moderate cognitive impairment, and less than 10 indicates severe cognitive impairment (25). The MMSE demonstrated good reliability, with the intraclass correlation coefficient for inter-rater reliability reaching 0.99, and the test–retest reliability was 0.91 (26).
4. University of California at Los Angeles Loneliness Scale (UCLA-LS): The UCLA-LS consists of 20 items designed to evaluate an individual’s feelings of loneliness. All items are measured on a 4-point Likert scale (ranging from “never” to “always”), and some items are scored in reverse. The higher the total score, the more severe the loneliness (27). Cronbach’s *α* of UCLA-LS was 0.92 (28).
5. Geriatric Depression Scale (GDS-15): The GDS-15 is used to assess depressive symptoms in older adults over a week and consists of 15 items. Responses indicating depressive symptoms are scored as 1 point, while other responses are scored as 0. A higher score on the scale reflects more severe depressive symptoms. The Chinese version of the scale has demonstrated good reliability and validity among Chinese older adults. Cronbach’s *α* of the GDS-15 (Chinese version) was 0.763 (29).
6. Summary of Diabetes Self-Care Activities Scale (SDSCA): The SDSCA is a widely used tool to measure diabetes self-management behaviors. It comprises 13 items and 6 subscales of diet, exercise, medication, blood glucose monitoring, foot care, and smoking. Except for smoking, each item is scored on a scale of 0–7 points, and each dimension was scored as the average score for that dimension, with higher scores indicating better self-management behaviors. Cronbach’s *α* of each dimension in SDSCA was 0.62–0.92 (30).
7. Family Adaptation, Partnership, Growth, Affection, Resolve index (APGAR): This assessment tool evaluates family function from five aspects (five items): family fitness, cooperation, length, emotion, and intimacy. The three responses, “almost always,” “sometimes,” and “almost never,” correspond to 3–1 points, respectively (31). A higher total score suggests better family function. The test–retest reliability coefficient was 0.83 (32).
8. Social Support Rating Scale (SSRS): The SSRS questionnaire is used to assess the level of social support for an individual, including three dimensions and 10 items, which mostly uses a 4-level Likert scale. The higher the total score, the higher the level of social support (33). The internal consistency across items of SSRS was 0.89–0.94, and test–retest reliability was 0.92 (34).
9. Impact on Participation and Autonomy (IPA) Questionnaire: The IPA questionnaire is used to measure the level of an individual’s social participation with good reliability and validity, including four dimensions and 25 items. The higher the total score, the less social participation. Cronbach’s *α* of each dimension in IPA was 0.81–0.91 (35).

### 2.3 Data collection method

Older adults with T2DM were recruited from the outpatient or inpatient departments of the hospitals or the community health service center. The researchers introduced the purpose and content of the study to the older adults and issued the questionnaire after obtaining their oral consent. At first, the cognitive function of the older adults was evaluated using MMSE; those who scored an MMSE score of >20 points continued to complete the questionnaire. The older adults without cognitive impairment completed the questionnaire by themselves, while the caregivers assisted those with mild cognitive impairment. If the participant had difficulties with reading, writing, or handwriting, the researcher would read the questions and complete the questionnaire based on the participant’s answers. After the questionnaire was completed, it was collected and checked on the spot. If there was an obvious omission or incorrect filling, a timely follow-up and improvement of the questionnaire were conducted. The questionnaire with 10% missing items or logical errors was deemed invalid. In this study, 508 questionnaires were distributed, and 12 invalid questionnaires were excluded. Finally, a total of 496 questionnaires were included, with an effective return rate of 97.64%, and the mean was used to interpolate missing values.

### 2.4 Statistical analysis and path analysis

Data were analyzed using Statistical Package for the Social Sciences (SPSS) version 26.0 and Mplus 8.0 software. Count data were expressed by frequency and composition ratio (%), and the chi-squared test was used for comparison between groups. Measurement data were compared between groups using the Mann–Whitney U test. The Spearman rank correlation analysis was used to explore the correlation between social isolation and various factors; *p* < 0.05 was considered to be statistically significant.

Model fitting was performed on the hypothesis model using Bayesian estimation. The model parameters were evaluated based on the posterior prediction distribution, trace map, autocorrelation map, and potential scale reduction (PSR). The fitting effect was evaluated based on posterior prediction *p*-value (PPp), the closer to 0.5, the better the model fit, and if less than 0.05, the model fit is considered poor (17). The insignificant path was adjusted to achieve the best-fitting effect to analyze the pathways through which different factors affect social isolation.

## 3 Results

### 3.1 Sample characteristics and social isolation status

A total of 496 older adults with T2DM were 60–91 years old, with a mean age of 68.88 ± 5.81 years; 273 males and 223 females. Their T2DM duration was 1–47 years, with a mean of 14.35 ± 8.69 years. There were 296 participants without cognitive impairment and 200 with mild cognitive impairment. The total score for LSNS-6 was (13.15 ± 5.61), of which 227 had social isolation, with an incidence of 45.77%. Seventy-four participants (14.92%) had social isolation at the family dimension and 239 (48.19%) at the friend dimension.

Univariate analysis was performed to evaluate the variables associated with social isolation in older adults with T2DM. There were significant differences in gender, age, educational background, marital status, working status, self-rating health status, and complications between the social isolation group and the non-social isolation group (Table 1).

**Table 1:** Comparison of older adults with T2DM having social isolation and no social isolation.

Subject	Group	No social isolation	Social isolation	*Z/χ* ^2^	*p*
Gender	Man	162	111	6.380	0.012
	Woman	107	116		
Age (years old)	60–69	181	102	27.980	0.000
	70–79	83	109		
	≥80	5	16		
Education background	Junior high school and below	71	74	10.023	0.007
	Senior high school/Vocational school	88	91		
	College and above	110	62		
Marital status	Having partner	253	194	10.202	0.001
	No partner	16	33		
Working status	Non-on-the-job	255	225	7.371	0.007
	On the job	14	2		
Self-rating health status	Poor	26	98	90.570	0.000
	Normal	148	106		
	Good	95	23		
Complications	No	198	118	24.897	0.000
	Yes	71	109		
Frequency of Internet use	Everyday	238	111	94.465	0.000
	At least once a week	16	75		
	At least once a month	4	17		
	Never	11	24		
Going-out frequency within a month	Everyday	212	82	108.159	0.000
	At least once a week	52	87		
	At least once a month	4	41		
	Never	1	17		
Number of chronic diseases		2.75 (2.00, 3.00)	3.01 (2.00, 4.00)	−2.054	0.040

### 3.2 Correlation analysis between social isolation and other factors in older adults with T2DM

It was shown that the occurrence of social isolation was negatively related to MMSE score, SDSCA exercise-management dimension score, smoking, APGAR score, SSRS score, visual impairment, hearing impairment, ADLs score, UCLA-LS score, GDS score, SDSCA diet-management dimension score, foot-care dimension score, and IPA score; but it was not related to community health service utilization, SDSCA blood glucose monitoring dimension score, and medication management dimension score (Table 2).

**Table 2:** Correlation of study variables.

Variable	Social isolation	Visual impairment	Hearing impairment	Health service utilization	ADLs	MMSE	UCLA-LS	GDS	Diet	Exercise	Blood glucose monitoring	Foot	Medicine	Smoking	APGAR	SSRS	IPA
Social isolation	1																
Visual impairment	0.345^a^	1															
Hearing impairment	0.222^a^	0.272^a^	1														
Health service utilization	−0.047	0.108^b^	−0.022	1													
ADLs	0.523^a^	0.293^a^	0.225^a^	−0.041	1												
MMSE	−0.405^a^	−0.437^a^	−0.228^a^	−0.062	−0.456^a^	1											
UCLA-LS	0.535^a^	0.162^a^	0.124^a^	−0.113^b^	0.337^a^	−0.059	1										
GDS	0.514^a^	0.280^a^	0.195^a^	−0.105^b^	0.454^a^	−0.406^a^	0.614^a^	1									
Diet	0.074^a^	0.050	0.052	0.110^b^	0.036	0.036	0.145^a^	0.048	1								
Exercise	−0.275^a^	−0.024	−0.135^a^	0.077	−0.328^a^	0.101^b^	−0.333^a^	−0.345^a^	0.112^b^	1							
Blood sugar monitoring	0.082	−0.031	0.115^b^	−0.133^a^	0.122^a^	0.139^a^	0.328^a^	0.132^a^	0.315^a^	−0.091^b^	1						
Foot care	0.094^b^	0.092^b^	0.052	0.117^a^	0.062	0.020	0.105^b^	0.099^b^	0.212^a^	0.102^b^	0.190^a^	1					
Medicine	0.027	0.113^b^	0.079	−0.040	0.057	−0.080	−0.148^a^	−0.039	0.043	0.104^b^	0.064	0.200^a^	1				
Smoking	−0.170^a^	−0.062	−0.056	−0.119^a^	−0.084	0.081	−0.009	−0.097^b^	−0.155^a^	−0.003	−0.061	−0.062	−0.001	1			
APGAR	−0.431^a^	−0.216^a^	−0.063	−0.008	−0.303^a^	0.373^a^	−0.416^a^	−0.542^a^	−0.005	0.187^a^	0.043	−0.048	0.017	−0.017	1		
SSRS	−0.710^a^	−0.314^a^	−0.235^a^	0.019	−0.421^a^	0.404^a^	−0.464^a^	−0.520^a^	−0.027	0.276^a^	−0.010	−0.085	−0.019	0.131^a^	0.511^a^	1	
IPA	0.651^a^	0.334^a^	0.252^a^	−0.010	0.624^a^	−0.483^a^	0.345^a^	0.557^a^	0.060	−0.277^a^	0.021	0.070	0.069	−0.123^a^	−0.441^a^	−0.645^a^	1

^a^*p* < 0.01; ^b^*p* < 0.05.

### 3.3 Path analysis model

**Figure 1:**
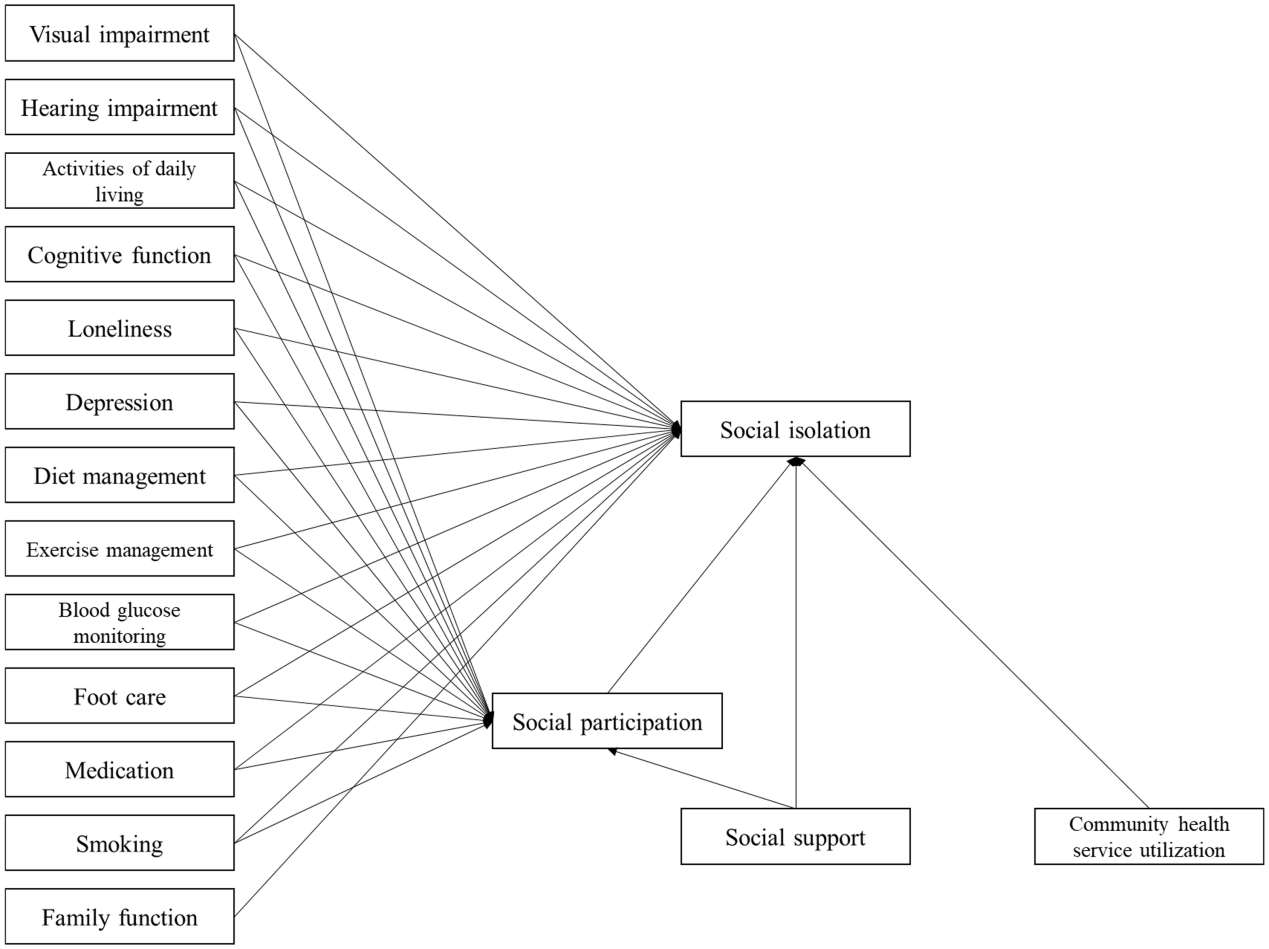
Hypothesized model of social isolation in older adults with T2DM.

**Figure 2:**
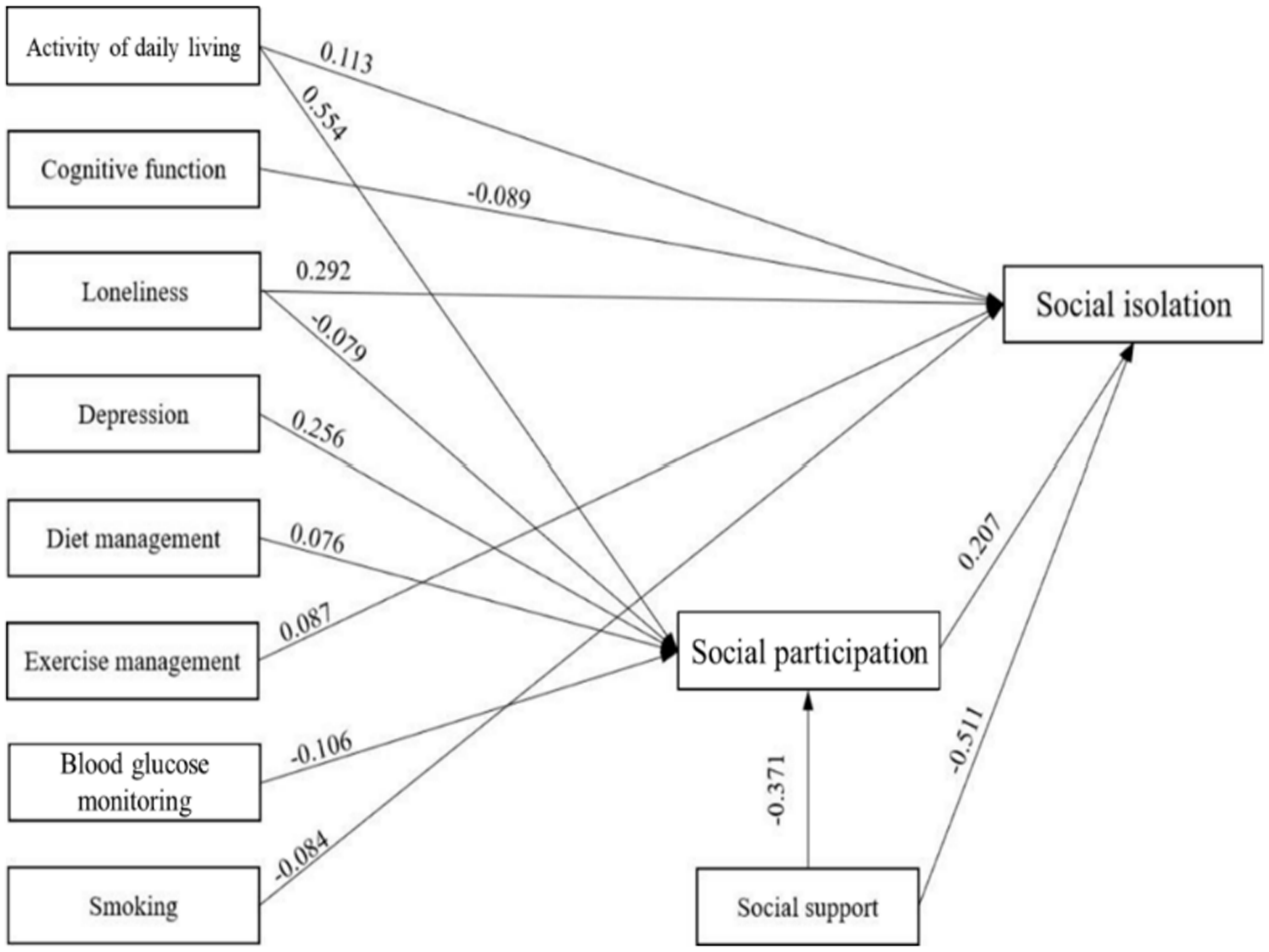
Path analysis model of social isolation in older adults with T2DM.

On the basis of the hypothesized path, with the occurrence of social isolation as endogenous variable, gender, age, education background, marital status, working status, self-rating health, complications, internet use frequency, number of chronic diseases as control variables, visual impairment, activity of daily living and other factors as exogenous variables, social participation as the mediator, the initial hypothesized model was established (Figure 1), and then model fitting was performed. The hypothesized model is shown to fit poorly. Non-significant paths were deleted, and the hypothesized model was corrected. Variable assignments of the model are shown in Table 3. The final model parameters exhibit good convergence and good fit (PPp = 0.071 > 0.05). The model shows the paths with statistically significant coefficients (Figure 2).

**Table 3:** Assignment table of factors affecting social isolation in older adults with T2DM.

Variable	Description
Social isolation	No = 0, Yes = 1
Visual impairment	No = 0, Yes = 1
Hearing impairment	No = 0, Yes = 1
Activity of daily living	Score of ADLs
Cognitive function	Score of MMSE
Depression	Score of GDS
Diet management	Average value of SDSCA diet management dimension
Exercise management	Average value of SDSCA exercise management dimension
Blood glucose monitoring	Average value of SDSCA blood glucose monitoring dimension
Foot care	Average value of SDSCA foot care dimension
Medication management	Average value of SDSCA medication management dimension
Smoking	No = 0, Yes = 1
Community health service utilization	No = 0, Yes = 1
Family function	Score of APGAR
Loneliness	Score of UCLA-LS
Social support	Score of SSRS
Social participant	Score of IPA

The direct, indirect, and total effects of factors on social isolation in older adults with T2DM are listed in Table 4. Cognitive function, SDSCA exercise management, and smoking only directly affect social isolation, while depression, SDSCA diet management, and blood glucose monitoring indirectly affect social isolation completely through social participation. Activity of daily living, loneliness, and social support not only directly affect social isolation but also influence it through social participation, with intermediary effect ratios of 50.00, 5.43, and 12.95%, respectively.

**Table 4:** Path analysis results of social isolation in older adults with T2DM.

Path	Effect type			Mediating effect ratio (%)
	Direct	Indirect	Total	
Social isolation ← Activity of daily living	0.113^b^	0.114^a^	0.228^a^	50.00
Social isolation ← Cognitive function	−0.089^b^	−0.009	−0.098^a^	—
Social isolation ← Loneliness	0.292^a^	−0.015^b^	0.276^a^	5.43
Social isolation ← Depression	−0.029	0.052^b^	0.024	100.00
Social isolation ← Diet management	0.016	0.015^a^	0.032	100.00
Social isolation ← Exercise management	0.087^b^	0.006	0.094	—
Social isolation ← Blood glucose monitoring	−0.037	−0.021^a^	−0.059	100.00
Social isolation ← Smoking	−0.084^b^	0.003	−0.081^b^	—
Social isolation ← Social support	−0.511^a^	−0.076^a^	−0.587^a^	12.95
Social isolation ← Social participation	0.207^a^	—	0.207^a^	—

^a^*p* < 0.01; ^b^*p* < 0.05.

## 4 Discussion

Based on Wenger’s conceptual framework of social networks for older adults, this study used a Bayesian method to test a structural equation model to determine the path of the influencing factors to social isolation in older adults with T2DM. Among 496 older adults with T2DM, 227 (45.8%) reported developing social isolation. Activity of daily living, cognitive function, loneliness, exercise management, smoking, social support, and social participation were directly associated with social isolation. In addition, six factors—activity of daily living, loneliness, depression, diet management, blood glucose monitoring, and social support—were indirectly associated with social isolation through their impact on social participation. Our research broadens the theoretical research field of social isolation in older adults with T2DM and provides a theoretical basis for formulating targeted interventions.

In this study, 45.77% older adults with T2DM experienced social isolation, which is higher than 22.7% of older adults with T2DM in Beijing in 2019 (6), which may be related to the reduction of going out and social interaction in older adults after the coronavirus disease 2019 (COVID-19) outbreak (36). It is also higher than 24.3% for community-dwelling older adults during the COVID-19 outbreak (37), indicating that the impact of the epidemic on the social network of older adults with T2DM may be higher than that of the general older adult population. Older adults with T2DM tend to reduce communication with the outside world to avoid infection or glucose fluctuations, which may lead to social isolation.

We found that the occurrence of social isolation in the friend dimension in older adults with T2DM was significantly higher than that in the family dimension (48.19% vs. 14.92%), which was consistent with the results of other studies of social isolation in older adults (38, 39). In general, personal family networks gradually stabilize from adolescence, while the size of friend networks gradually decreases in adulthood (40). In the context of Chinese “filial piety culture” (a Confucian value emphasizing children’s obligation to respect and care for aging parents), the majority of older adults with T2DM live with family members such as spouses or children. In this study, 473 participants (95.36%) lived with their families. Even among those who live alone, regular contact is maintained through visits or communication via telephone, WeChat (a widely used social app in China), and other approaches. As a result, social isolation in the family dimension occurs less (41). However, due to retirement and the demands of glycemic control, especially after developing diabetic complications, older adults often experience reduced participation in social activities and a shrinking network of friends. As a result, social isolation is prone to occur in the friend dimension.

After controlling for gender, working status, complications, and number of chronic diseases, the older adults with T2DM with low-level activity of daily living, poor cognitive function, high-level loneliness and low-level social support were more likely to have social isolation, which is similar to the findings of studies in the general older adult population (42–44). Due to aging and diabetes, older adults with T2DM are prone to have impaired cognitive function, which affects their judgment of time and place and therefore affects their social communication ability (45). The older adults with loneliness and a low level of social support think that they are not close to others and cannot get support from the outside world, so they consciously alienate themselves from others. All of these may increase the risk of social isolation.

We found the occurrence of social isolation in older adults with T2DM was also directly related to diabetes self-management behaviors in exercise management and smoking; the older adults with a low exercise management level tended to suffer from social isolation. It was reported that 38.9% of the Chinese older adults with T2DM kept regular exercise (46), and they tended to engage in aerobic exercise mainly by walking, and more than half of them exercise alone (47). Moreover, due to diabetes complications (36.29% in this study), part of the older adults had no energy to participate in other social activities (48) after exercise. These situations may lead to difficulties in maintaining or even reducing the social activity level of older adults with T2DM, resulting in social isolation. In many social occasions in China, there is a habit of handing and receiving cigarettes. Smoking is often seen as a social means of building social relationships and then communicating with friends, and correspondingly, the risk of social isolation for smokers is reduced (49). However, smoking accelerates the occurrence of vascular distortion and complications, which leads to the deterioration of the condition (50). Therefore, it is not advocated that older adults with T2DM increase social interaction through smoking, and other suitable social means can be used instead, such as playing cards and square dancing (for those without diabetes complications).

In this study, we found social participation is a mediation variable, which is confirmed the initial hypothesis, and six factors (activity of daily living, loneliness, depression, diet management, blood glucose monitoring, and social support) strengthen or weaken its influence on the risk of social isolation through the intermediary of social participation. The lower the level of social participation among older adults, the less family participation, social contact, and interaction, and the higher the risk of social isolation (42). It is worth noting that for older adults with T2DM, diet management and blood glucose monitoring were not directly related to social isolation, but rather indirectly affected social isolation through their impact on social participation. DiNardo et al. reported that dietary restrictions were associated with social isolation in individuals with diabetes (51). Our study found that older adults with T2DM who adhered to more stringent dietary control regimens exhibited diminished social participation, while those demonstrating greater adherence to blood glucose monitoring showed increased social participation. These two factors, along with the other four factors, were related to social isolation through social participation. In China, dinner is an important way for people to socialize. However, the older adults with T2DM worry that taking dinner with others may destroy their diet control and cause abnormal blood glucose fluctuations. On the other hand, older adults are concerned about maintaining a low-sugar diet that is unsociable and consciously reduce social activities (52). As a result, individuals with good diet management may reduce social participation, and the risk of social isolation increases correspondingly. Older adults who frequently monitor their blood glucose may be able to adjust their activity plan in a timely manner according to their blood glucose status, which can increase their confidence in participating in social activities and indirectly reduce the risk of social isolation (53).

Variables such as activities of daily living, loneliness, and social support are directly related to social isolation, and are also indirectly related to it through social participation. The scope and degree of social participation of older adults with good activities of daily living are not limited, and those with high-level social support are willing to participate in various social activities (54). However, we found that the older adults with high levels of loneliness had high levels of social participation. It may be because older adults with high levels of loneliness consciously increase family and social activities to reduce loneliness. Even if social participation behavior has increased, older adults still feel lonely. This kind of socializing is likely to be ineffective. In the next step, qualitative interviews should be conducted with the older adults with T2DM to understand the forms and real feelings of their social participation, and corresponding interventions need to be developed to reduce social isolation in this population.

It is recommended that community healthcare providers should prioritize older adults with T2DM who exhibit impaired activities of daily living, poor cognitive function, and high levels of loneliness, with regular assessment of their social isolation status. Given the mediating role of social participation, healthcare professionals should encourage social engagement among older adults while simultaneously guiding them to maintain dietary control and enhance self-monitoring of blood glucose levels. Considering both diabetes exercise management principles and the physical capabilities of older adults with T2DM, medical staff may organize group activities such as walking sessions or regularly arrange cultural and recreational programs, including calligraphy and music appreciation, thereby providing appropriate opportunities for social participation. Furthermore, it is advisable to improve supporting infrastructure that facilitates social engagement for older adults, creating more favorable conditions for their social activities.

## 5 Conclusion

This study showed a high prevalence of social isolation in older adults with T2DM and severe social isolation in the friend dimension. For older adults with T2DM, activities of daily living, cognitive function, loneliness, exercise, smoking self-management, and social support are directly related to social isolation. Social participation has a direct correlation with social isolation and is an intermediary factor between activities of daily living, loneliness, depression, diet management, blood glucose monitoring, self-management, social support, and social isolation. Depression, diet management, and blood glucose monitoring are related to social isolation completely through social participation.

## 6 Limitations

First, as this is a cross-sectional study, it does not allow for the determination of causal relationships between social isolation and the related factors. Second, the older adults with T2DM in this study were from Beijing, the capital city and a first-tier city in China, which may limit the representativeness of the sample to a certain extent. In the future, a multicenter follow-up study will be necessary to further reveal the mechanisms through which social isolation influences various factors in older adults with T2DM.

## Data Availability

The raw data supporting the conclusions of this article will be made available by the authors, without undue reservation.
