# Correction: Social isolation in older adults with type 2 diabetes mellitus—a path analysis

**DOI:** 10.3389/fpubh.2025.1674077

**Published:** 2025-08-29

**Authors:** Jing Wang, Xiaoyan Bai, Keke Lin, Chao Sun, Quanying Wu, Ruiting Zhang, Yu Liu

**Affiliations:** ^1^School of Nursing, Beijing University of Chinese Medicine, Beijing, China; ^2^Nursing Department, Beijing Hospital, National Center of Gerontology, Institute of Geriatric Medicine, Chinese Academy of Medical Sciences, Beijing, China; ^3^School of Traditional Chinese Medicine, Beijing University of Chinese Medicine, Beijing, China

**Keywords:** type 2 diabetes, aged, social isolation, influencing factors, path analysis

The title of this article was erroneously given as: “Social isolation in older adults with type 2 diabetes mellitus - a pathway analysis”. The correct title of the article is “Social isolation in older adults with type 2 diabetes mellitus—a path analysis”.

The original version of this article has been updated.

